# Design of experiments to compare the reprocessing effect with Fused Deposition Modeling printing parameters on mechanical properties of Polylactic Acid specimens towards circular economy

**DOI:** 10.1177/14777606221145702

**Published:** 2022-12-12

**Authors:** Tanay Kuclourya, Roberto Monroy, Rafiq Ahmad

**Affiliations:** Laboratory of Intelligent Manufacturing, Design and Automation (LIMDA), Department of Mechanical Engineering, 3158 University of Alberta, Edmonton, AB, Canada

**Keywords:** circular economy, reprocessing, Fused Deposition Modeling, raster angle, infill density, extrusion temperature, taguchi analysis

## Abstract

Distributed Recycling via Additive Manufacturing (DRAM) is a closed-loop material reprocessing solution that promotes circular economy. There are several literature gaps related to material properties and recycling cycles at different stages of the DRAM process. With an approach to filling these gaps, a small contribution has been made through this work by comparing the effect of reprocessing cycles (recycling) with the effect of FDM printing parameters such as Raster angle orientation, Infill density and Extrusion Temperature. These four parameters are ranked based on their impact on the tensile properties of Polylactic Acid (PLA) dog bone specimens. The Design of Experiments via Taguchi Analysis is carried out to avoid analysis of a large number of samples. The results show that recycling has the maximum impact on the tensile properties of PLA samples and can reduce the tensile strength by up to 75% in the course of four reprocessing cycles. The specimens had Ultimate Tensile Strength (UTS) values in the range of 20–26 MPa at the first reprocessing cycle which dropped significantly to a range of 7–9 MPa after the fourth reprocessing cycle. Additionally, a novel analysis on time and the number of specimens to be 3D printed at each reprocessing stage has also been conducted to help future researchers manage their printing schedule, especially in the recycling domain.

## Introduction

Recycling plastics is a necessary step towards the reduction of new plastic feedstock and minimizing the amount of energy required for its production.^1^ The process of plastic recycling gets difficult when it reaches the end of its life,^2^ and hence it is important to recycle them at an early stage in order to prevent their disposal in oceans and landfills. The main threat is not the usage of plastics, but the disposal of plastics after their use,^3^ and this generates an urgent need to develop mechanisms for recycling polymeric wastes economically and sustainably following the environmental safety and plastic waste management rules.^4^ A serious concern is that polymeric materials consume around 4% of the global production of oils and gas in the form of feedstock, whereas another 3–4% is used in their energy transformation.^5^ This deduces that it is important to use the polymeric materials efficiently in order to ensure minimal wastage.^6^ However, at the same time, it has been estimated that there is an annual consumption of 18,500 tons of plastics used in 3D printing.^7^ Out of this, almost 70% contributes to plastic waste and gets accumulated in the environment.^8^ This raises the need for ‘Circular Economy’, which makes the after-life use of plastics and contributes to the supply chain.^9^ Recycling plastic is one such action that promotes this strategy.^10^ The plastic circular economy promotes the flow of plastics in a closed cycle, which leads to a sustainable economy with optimized production costs and minimal plastic pollution.^11^ It tends to avoid harmful emissions and, at the same time, harness all the extraordinary properties of the plastic material.^12^

Distributed Recycling via Additive Manufacturing (DRAM) is one potential solution for improving sustainability and promoting a circular economy worldwide.^5^ 3D printing technologies have widened their applications to various fields because of their efficiency, precision, and accuracy ^13^ and have provided ways to utilize recycled plastics and convert them to useful items.^14^ However, despite having good material efficiency at a low level, the 3D printing process is still a threat as far as material sustainability is concerned.^15^ To handle this issue of sustainability, closed-loop recycling, which is a key to a circular economy,^16^ can be a potential measure that can restrict the need to explore more commercially viable materials for the 3D printing process as it is way ahead of other processing methods like down-cycling and to landfill.^15^

**Figure 1.**
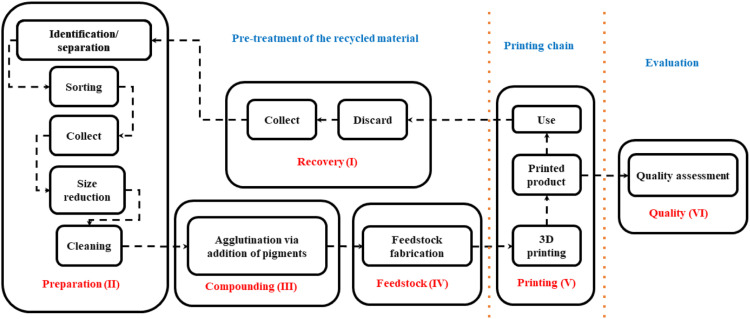
Closed loop recycling framework of DRAM (adapted from.)^17^

The closed loop DRAM chain comprises of six stages which are recovery, preparation, compounding, feedstock, printing and quality^18^ as shown in Figure 1. The recovery stage collects the plastic which gets sorted and identified in the preparation phase.^17^ The next step of preparing the sorted plastic into a form suitable enough to be used as a feed material is done in the compounding phase. This feed material can be either be a single plastic or a composite material.^17^ The advanced phase of this step is to prepare a recycled material which can be used as a feedstock for 3D printing. This is done in the feedstock phase.^18^ Once the feedstock is prepared, the printing phase takes care of the 3D printing.^17^ Lastly in the quality phase, the material quality check is done at the raw, feedstock as well the final 3Dprinted stage.^17^ Having discussed the concept of DRAM, it is essential to know that till date, there are several gaps in the DRAM literature. For instance, the printing parameters of a recycled material are still not defined in the printing phase. Only few materials have been tested for their recycling ability which leaves a huge literature gap in the feedstock phase. Additionally, there is still a lack of information on the material properties after recycling and its effect on the 3D printing process.

The feedstock materials used for 3D printing are fairly expensive and cost around 19-80 USD/kg.^18^ These high material costs also promote the concept of plastic recycling.^18^ Many extruders such as Felfil filament extruder, Filabot, Filafab, Protocycler^+^, 3Devo, Noztek, Robotdigg etc., have started utilizing both recycled and virgin pellets to produce filaments.^19–24^ On the other hand, organizations such as Plastic bank, ProjectSeafood, Perpetual Plastics Project etc., also work dedicatedly on waste plastic recycling for 3D printing filaments.^18^ Several studies have aimed toward developing and utilizing biodegradable and recycled filaments in 3D printing technologies.^25–27^ Utilizing recycled plastic wastes and transforming them into plastic filaments suitable for FFF (Fused Filament Fabrication) or FDM (Fused Deposition Modeling) printers has become a need of the current scenario.^18^

The literature signifies that FDM is one of the most critical methods which is associated with plastic recycling. It is a complex process as it is associated with multiple parameters while only a specific parametric combination yields optimum results.^28^ Many studies have been conducted in the past which have shown the influence of a specific set of parameters on the mechanical properties of the FDM printed parts.^29–31^ Likewise, the scope of this work is limited to analyzing the effect of FDM parameters - Infill Density (ID), Raster Angle (RA), and Extrusion temperature (ET) on the tensile strength of virgin and up to 3 times recycled Polylactic Acid (PLA). As per the literature, RA is a critical FDM parameter that can be defined as the direction of roads (beads material) relative to the direction of loading of the part.^32^ RA governs the anisotropy of the printed product.^33^ It has been found that longitudinally aligned rasters display higher tensile strength than transversally aligned raster orientation.^34^ There are typically four types of raster angles- 0° or axial, 45°/−45° or crisscross, 0°/90° or cross, and 90° or transverse.^35^ The majority of the studies have been conducted for these values of raster angles which signifies a gap in the literature. ID is yet another parameter that plays a critical role in ensuring a good bonding strength between the rasters and the layers.^36^ This parameter indicates the quantity of material with which the component is 3D printed.^37^ The modulus and the tensile strength of the FDM product are significantly affected by the ID.^38^ Low ID contributes to low load-bearing capacity, whereas high ID contributes toward higher tensile strength.^39^ Hence to obtain low weight and high strength for a product, it becomes necessary to have an optimum ID.^40^ Lastly, the third FDM parameter considered in this work is the ET. Since the FDM process is prone to thermal degradation, it involves high temperature, which often leads to a loss of viscosity in the filaments by virtue of increased fluidity.^41^ Hence an over-excessive temperature can lead to void generation in the final printed product, which attributes to low strength and dimensional inaccuracy.^42,43^ On the other hand, if the ET is low, the material will not melt properly, eventually clogging the nozzle.^44^ It is, therefore, a critical parameter and has a governing role in the strength of the ABS and PLA fabricated parts.^45^ Although PLA has a linear relation with the tensile strength for a temperature range of 200–220°C,^46^ literature shows that studies have failed to form a generic relationship between the ET and the tensile strength.

FDM processes generally demand materials that have bulk strength and elastic moduli in the range of 30–100 MPa and 1.3–3.6 GPa, respectively.^47^ The material properties govern the type of recycling process to be used for plastics.^48^ Some common thermoplastics, such as PLA and ABS, are mainly treated by physical recycling methods.^11^ These thermoplastics are first shredded and reprocessed after melting.^13^ ABS has excellent properties of heat resistance, high impact resistance, and toughness.^49^ 3D printing, when done with ABS, generates harmful fumes of ultra-fine aerosols.^50^ However, despite being a very common 3D printing thermoplastic, there are varying findings associated with the properties of ABS and hence there is a requirement for more studies to be conducted in order to utilize ABS for widespread applications.^51^ On the other hand, PLA is a linear aliphatic thermoplastic polyester that is extracted from natural sources and has superior thermos-physical properties.^52^ High brittleness and poor thermal stability are some of the few demerits which limit its uses.^53^ Since PLA is biodegradable, it is often preferred over other printing materials as it does not contaminate the environment upon degradation.^49^ As far as the scope of this work, PLA has been used to compare the abovementioned mechanical properties. This will establish a base study for other potential thermoplastics such as ABS, PC, HIPS, etc.

Recycled material is cheaper than new virgin material, brings less energy consumption, and is environmentally friendly as the carbon footprint is reduced by at least 80%.^54^ However, when it comes to utilizing recycled materials for 3D printing, it should be noted that many 3D printing technologies still lack information on the mechanical properties of reprocessed materials, and to increase the viability of using recycled materials for 3D printing purposes, there is a need for profound analysis in terms of material defects, processing conditions, end quality, and performance properties after recycling.^55^ Hence, a novel idea of including the ‘number of reprocessing cycles’ as the fourth influencing factor has been considered in this work. In this way, this work aims to serve as a base study for filling several literature gaps of the DRAM approach, which are discussed earlier in this section. Finally, using the Design of Experiments - Taguchi Analysis, the four parameters have been ranked based on their severity in affecting the tensile strength of PLA printed ASTM standard D-638 Type 1 tensile specimens. The results have been analyzed, and suitable inferences have been derived.

## Experimental details

This section provides an in-depth idea of various experimental aspects such as the material used, FDM process parameters, machines used, ASTM standard used, speed test, execution of design of experiments, and the time analysis.

### Materials

As mentioned in the earlier section, PLA has been used in this work. The commercial 3D printing PLA filaments have been acquired from Innofil^3D^, which is a Canadian filament store. The standard extrusion temperatures have been maintained to avoid the warping and stringing issues during printing. Similarly, an optimum bed temperature well above the material’s glass transition temperature has been maintained to ensure a reduced surface tension between the material and the bed surface, which leads to proper adhesion.^56^ Lastly, in order to avoid inconsistencies in the print and jamming of the FDM extruders, it is necessary to remove any possible atmospheric moisture absorbed by the filaments when exposed to the environment.^57^ Hence, the filaments are dried at a specific temperature for a specific time, depending on the material type. The specifications for PLA are shown in Table 1.

**Table 1.** Material specifications.^58^

Material	Filament diameter	Standard extrusion temperature range	Standard bed temperature rangeact	Drying temperature, °C	Drying time
PLA	1.75 mm	210–230°C	50–70°C	80	4 hours

### Methodology

**Figure 2.**
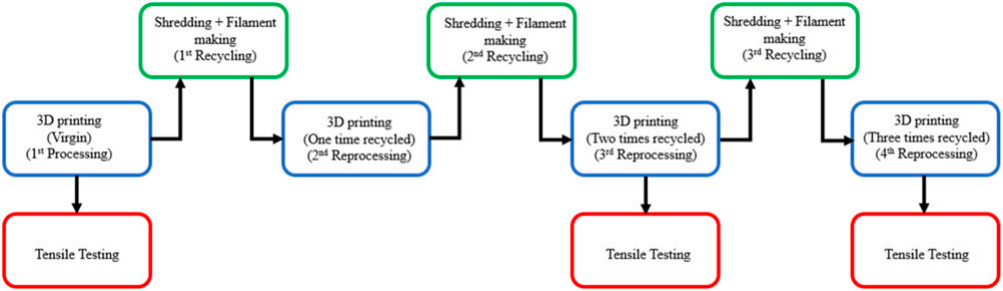
Research methodology flowchart- Taguchi Analysis I.

**Figure 3.**
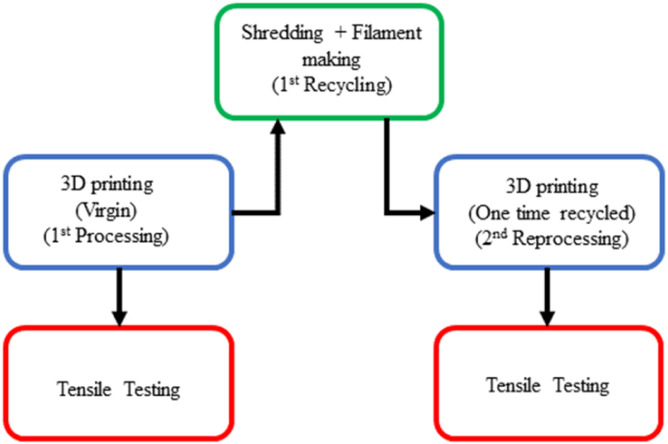
Research methodology flowchart- Taguchi Analysis II.

This section discusses in detail the methodology followed in this work. The flowcharts shown in Figures 2 and 3 describe the entire process of printing, shredding, filament making, reprocessing, and tensile testing of specimens.

## Design of experiments and Taguchi Analysis

As already discussed, four controllable factors (Infill density, Raster angle, Extrusion temperature, and Number of reprocessing cycles) have been identified that can affect the tensile strength of the 3D printed specimens. Taguchi’s Design of experiment method has been used for ranking these four parameters based on the severity they have in affecting the tensile properties of the specimens. While designing experiments, this method considers the effect of the parameters on the mean and variance of the process performance characteristics, which is a check of the well-functioning of the process. This method makes it possible to analyze the effect of multiple parameters with very few tests with the help of orthogonal arrays. The parameters are organized in such a way that minimum possible tests have to be conducted instead of conducting all the test combinations in the factorial design. Through this technique, it becomes possible to analyze the most significant factor which affects the product quality with minimum experimentation and saves plenty of time and resources.

The size of the orthogonal array is determined by the number of parameters and the number of levels. In Taguchi analysis, the level of a parameter signifies the value of that entity. On increasing the number of levels, the Taguchi experiment size increases as the parameters are then assessed at a greater number of values. The unit of levels might change as per the parameter. For example, in Table 2, the levels 1, 2, and 3 for infill density are 30°, 60°, and 90°, respectively. In this work, conventional values are chosen for all the levels of each parameter. This is done to ensure that enough literature is present to validate the trends in the tensile test results of the specimens. For example, in case of raster angle, cross orientation (0°/90°), crisscross orientation (45°/−45°) and 30°/−60° orientations have been chosen. Likewise, conventional values have been selected for levels of infill density and extrusion temperature. In this work, PLA has been reprocessed four times, hence the values of levels for the reprocessing parameter have been selected in a way that all the reprocessing cycles are covered in both the Taguchi analyses (1, 3 and 4 in TAI whereas 1 and 2 in TAII.)

**Table 2.** Parameters and Control levels as per Taguchi L-9 array.

Experiment number	A (raster angle)	B (infill density)	C (extrusion temperature)	D (number of reprocessing cycles)	Specimen nomenclature (used in this work)	Tensile strength (X)
1	1 (0°/90°)	1 (30%)	1 (200°C)	1 (1)	P.0.1.3.1	X1
2	1 (0°/90°)	2 (60%)	2 (210°C)	2 (3)	P.0.2.6.3	X2
3	1 (0°/90°)	3 (90%)	3 (220°C)	3 (4)	P.0.3.9.4	X3
4	2 (30°/−60°)	1 (30%)	2 (210°C)	3 (4)	P.3.2.3.4	X4
5	2 (30°/−60°)	2 (60%)	3 (220°C)	1 (1)	P.3.3.6.1	X5
6	2 (30°/−60°)	3 (90%)	1 (200°C)	2 (3)	P.3.1.9.3	X6
7	3 (45°/−45°)	1 (30%)	3 (220°C)	2 (3)	P.4.3.3.3	X7
8	3 (45°/−45°)	2 (60%)	1 (200°C)	3 (4)	P.4.1.6.4	X8
9	3 (45°/−45°)	3 (90%)	2 (210°C)	1 (1)	P.4.2.9.1	X9

As per Taguchi, product quality attained is maximum when the product is immune to uncontrollable environmental factors and the deviation from the target value is the minimum. This ratio of product quality (signal) and the uncontrollable factors (noise) is termed as Sn ratio (Signal/noise ratio). To achieve good product quality, Sn ratio should be high. It is mathematically given as –

$$SNi=10\log{\frac{yi}{si^{2}}}^{2}$$

Here in the above equation, SNi denotes the signal-to-noise ratio of the i^th^ experiment. yi and $si^{2}$ denote the average and variance of all the data values of the i^th^ experiment.^59^

However, there are several disadvantages of the Taguchi method. Firstly, the Taguchi analysis is relative; hence, it does not provide an exact conclusion as to which parameter has the highest effect on performance characteristics. Secondly, the orthogonal array does not consider all the variable combinations. This restricts its use at places where the interaction of variables is to be considered.^59^

In the present work, two different Taguchi analyses have been conducted for validation purposes. In Taguchi analysis-I (TA1) the four parameters have been investigated with three-level responses. As per Taguchi, for four parameter-three level analysis, a 9-run array or L-9 array should be selected**.**^60^ An L-9 array signifies that only 9 different parametric combinations need to be 3D printed and tested. These nine combinations are shown in Table 2.

From Table 2, the three levels of parameter A are 0°/90°, 30°/−60° and 45°/−45° raster angle configurations. The three levels of parameter B are 30%, 60% and 90% infill densities. Whereas for parameter C, the three levels are 200°C, 210°C and 220°C. Similarly, the three levels for parameter D are 1, 3, and 4 reprocessing cycles. However, it should be noted that parameter D is quite a complex parameter when it comes to the analysis of the total number of specimens to be 3D printed and the time taken to print them over four reprocessing cycles. It considers the efficiencies of the shredder and the filament maker, making the analysis a bit complex. Due to this efficiency issue, more specimens need to be printed in the initial stages as compared to the later stages.

Similarly, in the second Taguchi analysis (TA2), the four parameters were investigated with two-level responses. As per Taguchi, for four parameter-two level analysis, an 8-run array or L-8 array should be selected**.**^60^ An L-8 array signifies that only 8 different parametric combinations need to be 3D printed and tested. These eight combinations are shown in Table 3.

**Table 3.** Parameters and Control levels as per Taguchi L-8 array.

Experiment number	A (raster angle)	B (infill density)	C (extrusion temperature)	D (number of reprocessing cycles)	Specimen nomenclature (used in this work)	Tensile strength (X)
1	1 (0°/90°)	1 (30%)	1 (200°C)	1 (1)	P.0.1.3.1	X1
2	1 (0°/90°)	1 (30%)	1 (200°C)	2 (2)	P.0.1.3.2	X2
3	1 (0°/90°)	2 (90%)	2 (220°C)	1 (1)	P.0.2.9.1	X3
4	1 (0°/90°)	2 (90%)	2 (220°C)	2 (2)	P.0.2.9.2	X4
5	2 (45°/−45°)	1 (30%)	2 (220°C)	1 (1)	P.4.2.3.1	X5
6	2 (45°/−45°)	1 (30%)	2 (220°C)	2 (2)	P.4.2.3.2	X6
7	2 (45°/−45°)	2 (90%)	1 (200°C)	1 (1)	P.4.1.9.1	X7
8	2 (45°/−45°)	2 (90%)	1 (200°C)	2 (2)	P.4.1.9.2	X8

From Table 3, the two levels of parameter A are 0°/90°, and 45°/-45° raster angle configurations, for parameter B are 30% and 90% infill densities, whereas, for parameter C, the two levels are 200°C and 220°C. Lastly, the two levels for parameter D are one and two reprocessing cycles. The calculations of the experimental results using Taguchi Analyses I and II, alongwith the number and time analyses for specimens are shown in coming sections.

Figures 2 and 3 show the research methodology flowchart for TA1 and TA2, respectively. The tensile specimens are 3D printed as per the batch size required at each processing cycle. As per the number analysis, for TA1, tensile testing is conducted for three of the specimens at this first processing stage, and then the required number of specimens are shredded to get sufficient material to make filaments for printing the specimens for the second reprocessing cycle. This process continues, and tensile testing is conducted for the specimens of the third and the fourth reprocessing cycles, whereas for TA2, the process stops at the second reprocessing cycle. Hence to conclude, among TA1 and TA2, the former involves reprocessing effect at first (Virgin), third (two times recycled), and fourth (three times recycled) reprocessing cycle. In contrast, the latter involves the reprocessing effect at the first (Virgin) and second (one-time recycled) reprocessing cycle. For both TA1 and TA2 analyses, three specimens of each combination are tested in order to avoid uncertainty in the results.

## 3D printing of Specimens

**Figure 4.**
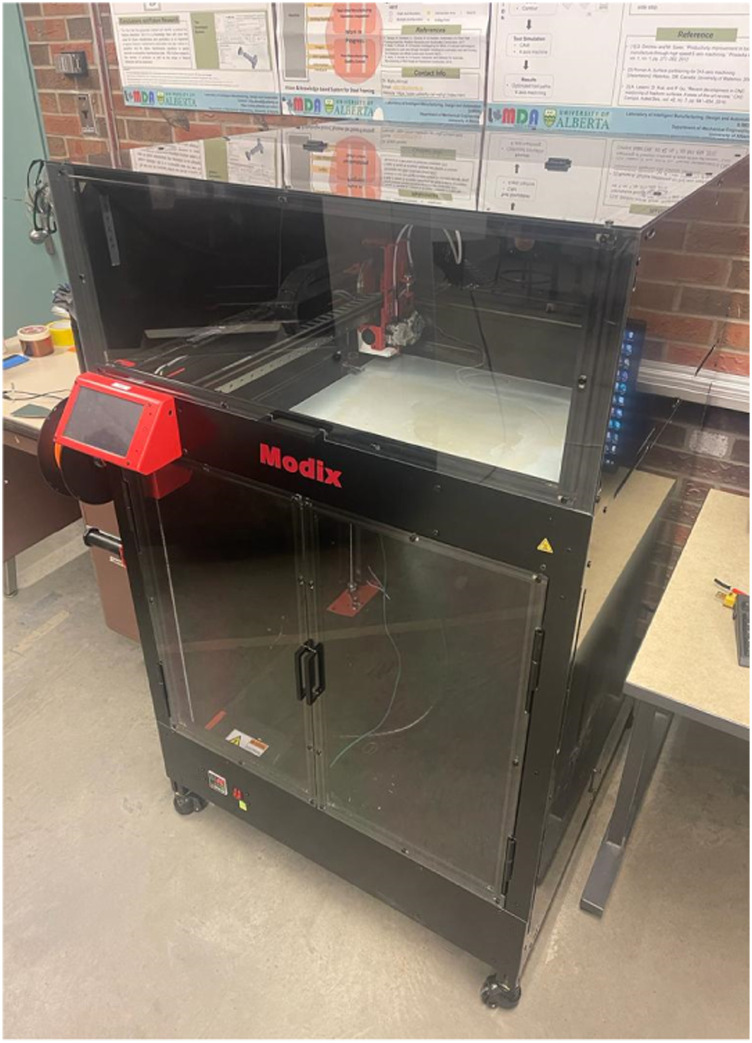
Modix 3D printer.

A large-scale 3D printer-Modix BIG-60 V3, shown in Figure 4, has been used in this work. It has a print volume of 600 X 600 X 660 mm (XYZ), making printing large-sized objects and multiple objects feasible. It leads to less material wastage and reduced printing time, which results in cheap printing costs.

Since this research aims to compare the effect of Infill density, Raster Angle, and Extrusion temperature along with a non-FDM parameter (number of reprocessing recycles) on the tensile strength of 3D printed PLA specimens, the other parameters are kept constant, and their most optimum values have been derived from the literature. The FDM parameters are mentioned in Table 4.

**Table 4.** Description of FDM parameters.

Parameter	Value	Source
Infill density	30%, 60%, 90%	—
Raster angle	0°/90°, 45°/−45°, 30°/−60°	—
Extrusion temperature	200°C, 210°C, 220°C	—
Infill pattern	Grid	^61^
Build orientation	Flat	^62^
Layer height	0.2 mm	^63^
Retraction distance	3.5 mm	Based on initial printing trials
Build plate adhesion type	Brim (width = 3 mm)	Based on initial printing trials

In this work, the layer height has been set at 0.2 mm, which makes the specimen a print of 16 layers. All the specimens in this work are printed using a 0.4 mm nozzle. Another important FDM parameters are the speed parameter. These parameters mainly include printing speed, traveling speed, and retraction speed. All these parameters have a direct relationship with the printing time. Traveling speed denotes the extruder’s motion when it is not extruding the filament. Although increasing the traveling speed significantly decreases the print time, an excessive speed might result in a ringing effect on the printed part.^64^ For this work, the traveling speed has been set at 80 mm/s. On the other hand, Retraction speed signifies how quickly an extruder pulls back the filament just before traveling. It plays an important role as higher retraction speeds can result in stringing issues, whereas lower speeds can lead to the generation of blobs within the print.^65^ A retraction speed of 25 mm/s has been adopted for this work. Lastly, Printing speed depicts the motion of the axes motors as well as the extruder motors. Low printing speed forces the nozzle to rest on the printed plastic layer for more than the required time, which results in print deformation. In contrast, excessive printing speed may result in insufficient cooling, ringing issues, and weak interlayer adhesion.^66^ Hence it is important to have an optimum printing speed to ensure a good quality product.

**Figure 5.**
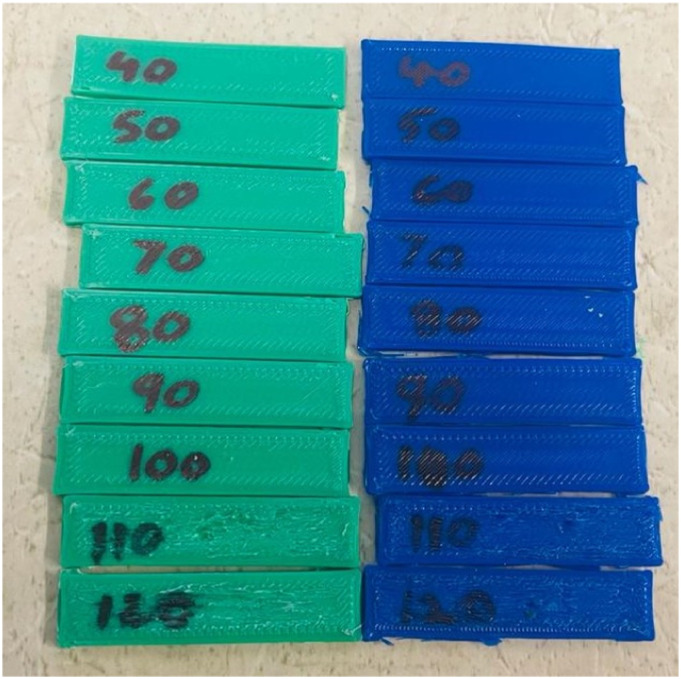
Speed test specimens (Green - virgin PLA, Blue – one-time recycled PLA).

For this, a print speed test was conducted on both virgin and recycled PLA specimens. A test specimen of dimension 10 X 40 X 2 mm was designed and printed at speeds of 40, 50, 60,70, 80, 90, 100, 110 and 120 mm/s. It can be seen from Figure 5 that at speeds above 100 mm/s, there were issues of layer shifting in the test specimens. Hence all the tensile specimens have been printed at a printing speed of 100 mm/s in this work. Separate tests were conducted for two- and three-times recycled PLA and 100 mm/s was again found to be the optimum speed.

## Tensile testing of specimens

**Figure 6.**
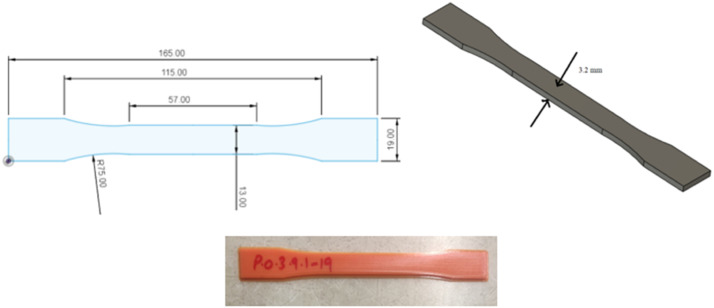
ASTM D638 Type I design designed tensile specimen and final 3D printed tensile specimens.

The tensile specimens are 3D printed as per the ASTM D638 (Type I) standard. The CAD model of this standard was designed on Autodesk Fusion 360 and is shown in Figure 6. This 3D CAD model is then exported to slicing software Ultimaker Cura. This software processes the part in STL file format, tessellates it into several basic triangular components, and further slices it into several horizontal sections. The FDM process then generates these two-dimensional contours and stacks them above each other.^67^ The final 3D printed PLA specimen is shown in Figure 6.

**Figure 7.**
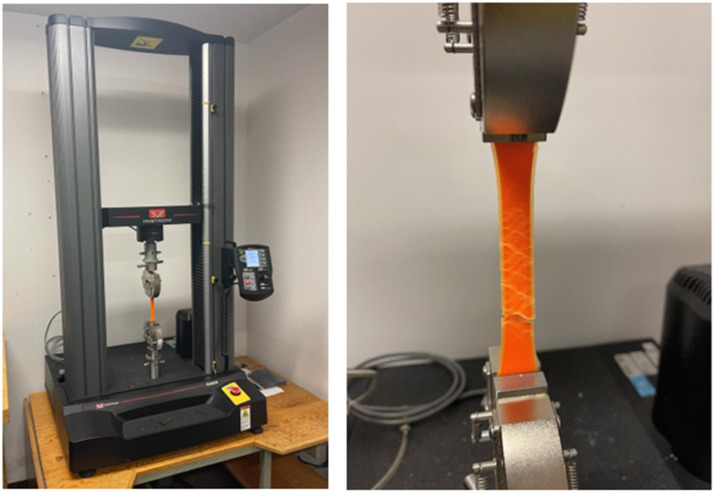
Instron 5966 machine used for tensile testing.

Once the specimens are 3D printed, tensile strength analysis of the specimens is done using Instron 5966 machine at a stroke rate of 2 mm/min. The machine uses a load cell of 10 kN and a gripper of a maximum load 5 kN. The Instron machine used in this work can be seen in Figure 7.

## Specimen shredding and filament making

**Figure 8.**
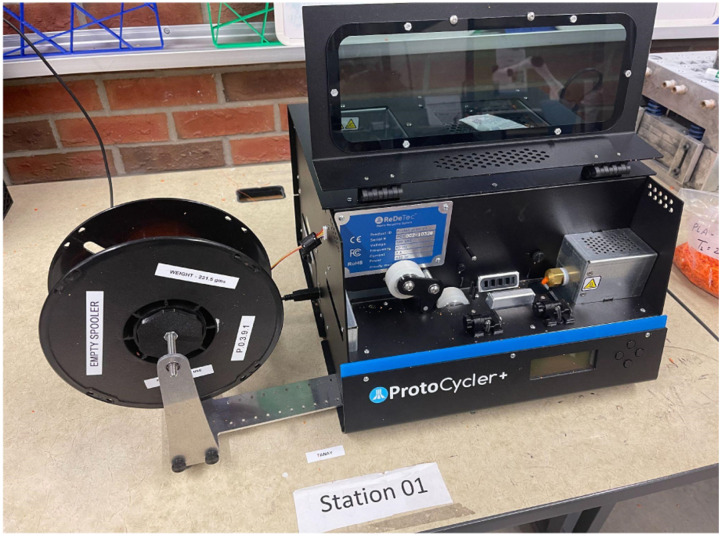
ProtoCycler + setup used for shredding and filament making.

**Figure 9.**
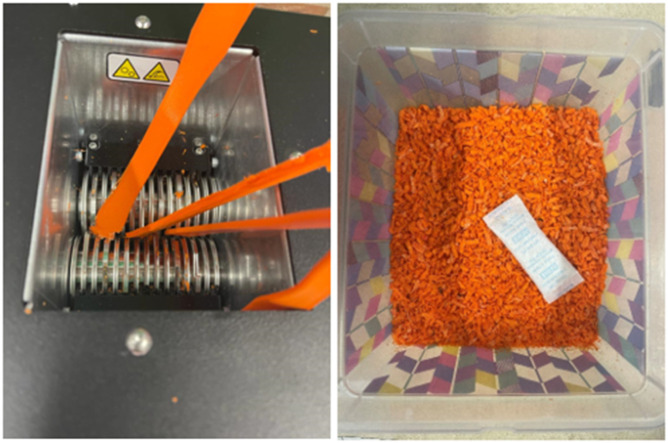
ProtoCycler + grinder and grinded PLA particles.

The specimens which are not undergone through tensile testing are shredded and made into filaments. The ProtoCycler + machine manufactured by ReDeTec, Canada, shown in Figure 8, has been used in this work. It is an advanced desktop extruder having arrangements for both the grinding and filament-making process. The grinder has a 32:1 gearing system which provides high torque to the extruder screw. To ensure good quality extrusion of filaments, the shredded particles’ size was kept between 3 mm–5 mm. The grinder as well as the grinded PLA particles, are shown in Figure 9. Uniform particle shape and size lead to good extrusion. After grinding, the shredded particles were dried and then fed to the extruder chamber. Shredded PLA specimens were dried at 80°C for 4 hours. ProtoCycler + has the capacity to extrude a maximum throughput of 500 grams per hour.

**Figure 10.**
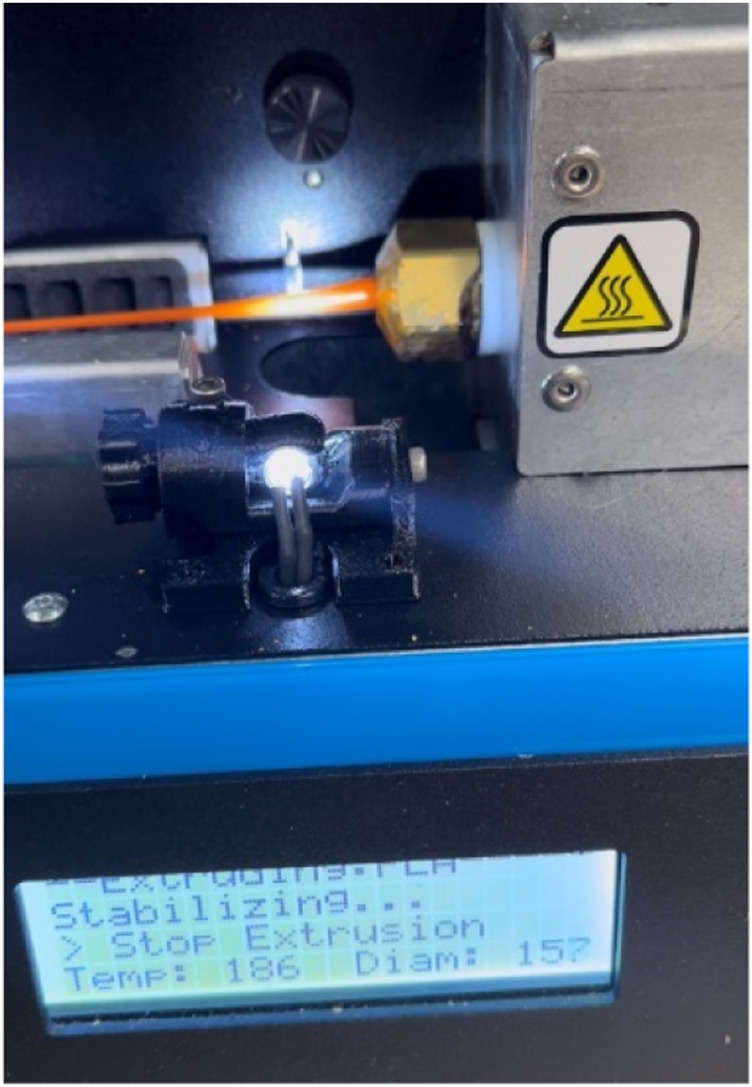
ProtoCycler + filament maker.

Also, the diameter of the extruded filament has a measurement precision of 0.01 mm. Although 1.75 mm diameter has been chosen as a standard measure for the extruded filaments in this work, the output diameter was in the range of 145–185 mm, which was suitable enough to be 3D printed using Modix. This device can be operated at a maximum temperature of 250°C. Figure 10 shows the filament getting extruded from the ProtoCycler + setup.

**Figure 11.**
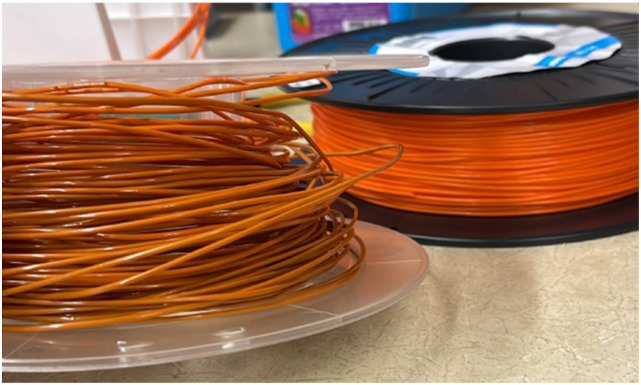
Color shift in PLA filament by virtue of recycling (left – filament made from two times reprocessed PLA material, right – virgin PLA filament).

An interesting observation during the process of filament make was the color shifting property of the material by virtue of mechanical and thermal degradation.^68^ Figure 11 shows the visible change in color of a virgin PLA filament as well as the filament made from two-time processed PLA material.

From various trials, it was estimated that for PLA, the filament maker mass efficiency was around 70% for virgin or one-time processed material, and this efficiency dropped by around 5% for every subsequent processing cycle. On the other hand, the grinder mass efficiency for PLA was found to be 89% for all the processing cycles. These efficiencies have a very crucial role in estimating the number of specimens which need to be 3D printed at every reprocessing stage and have been discussed elaborately later in this work.

## Analysis of results

### Specimen weight analysis

**Figure 12.**
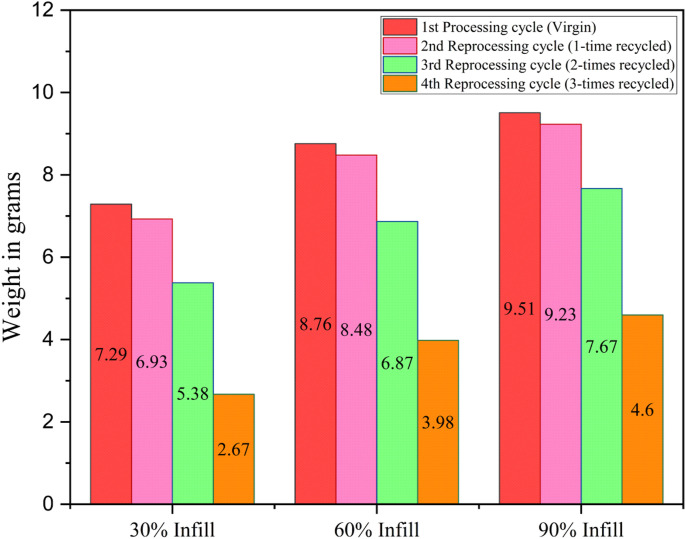
Average weight of specimens (in grams) as per the infill density-recycling stage combination.

Infill density signifies the amount of material present inside a 3D printed component.^17^ Hence more infill density naturally means more volume of material inside which means more weight of the specimens. For example, in this work, 90% infill specimens are heavier than 60% infill specimens followed by 30% infill specimens in respective reprocessing cycles. Now, recycling a plastic degrades its mechanical and rheological properties.^1^ Most thermoplastics often experience a drop in density under recycling.^69^ The level of degradation increases with every subsequent reprocessing cycle, which reduces the density at every cycle. Now density is the ratio of mass over volume and since in this work, the analysis was done on the dog bone specimens (constant volume), a drop in mass or weight was observed with the increase in reprocessing cycles. Figure 12 shows the specimens’ weight drop as per their infill density and reprocessing cycle combination.

From the results in Figure 12, it can be seen that the weight of the specimens had a significant drop consistently till the fourth processing cycle. Although the percentage drop varied for different infill densities, a similar pattern of increase in the percentage reduction of weight with increasing reprocessing cycles was common for all the infill density specimens. 30% of infill density specimens experienced a drop in weight by around 5%, 22%, and 50% with every increasing reprocessing cycle. In contrast, for 60% of infill specimens, the percentage reduction in weight was around 3%, 19%, and 42% for every subsequent reprocessing cycle. Lastly, for 90% infill density, the percentage drop was around 3%, 17%, and 40% for subsequent reprocessing cycles.

### Mechanical test results

**Figure 13.**
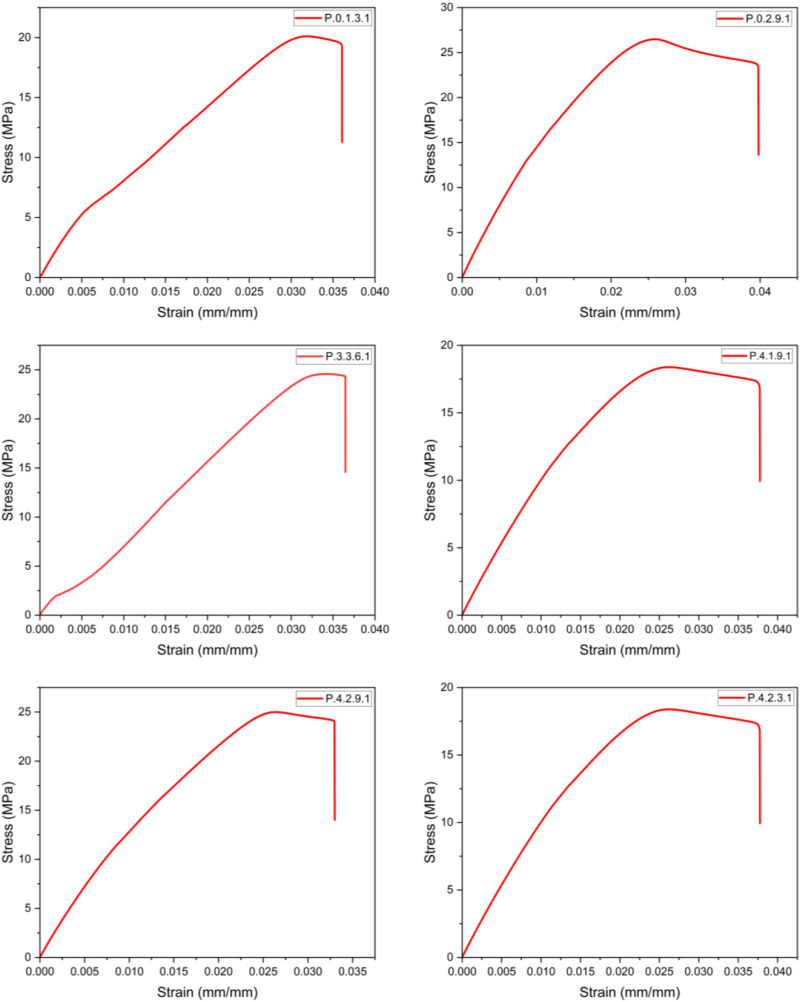
Stress-Strain analysis for specimens of 1st reprocessing cycle.

**Figure 14.**
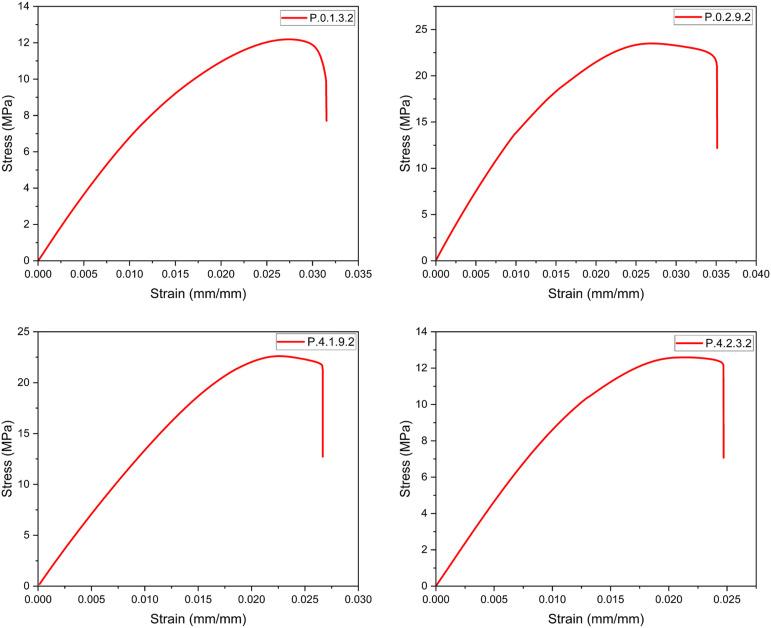
Stress-Strain analysis for specimens of 2nd reprocessing cycle.

**Figure 15.**
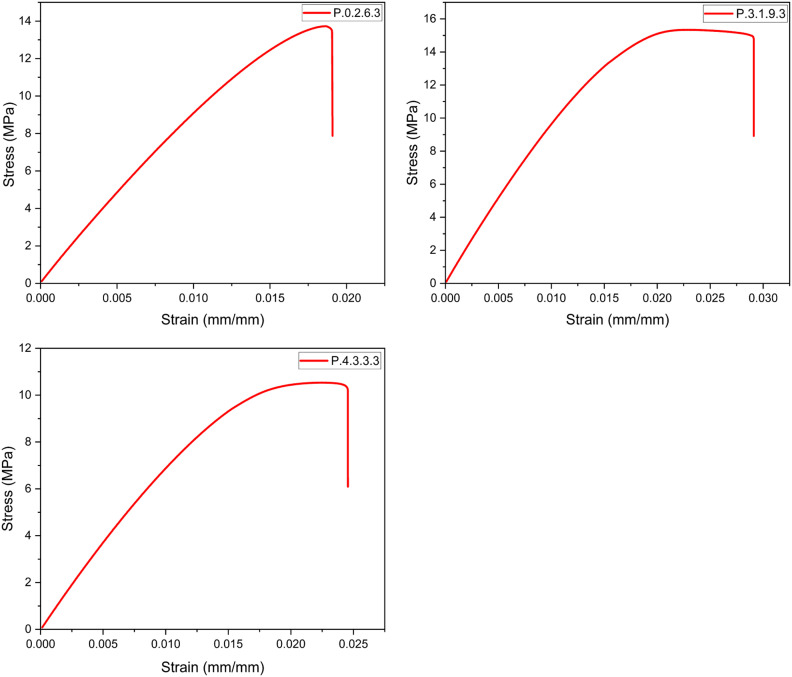
Stress-Strain analysis for specimens of 3rd reprocessing cycle.

**Figure 16.**
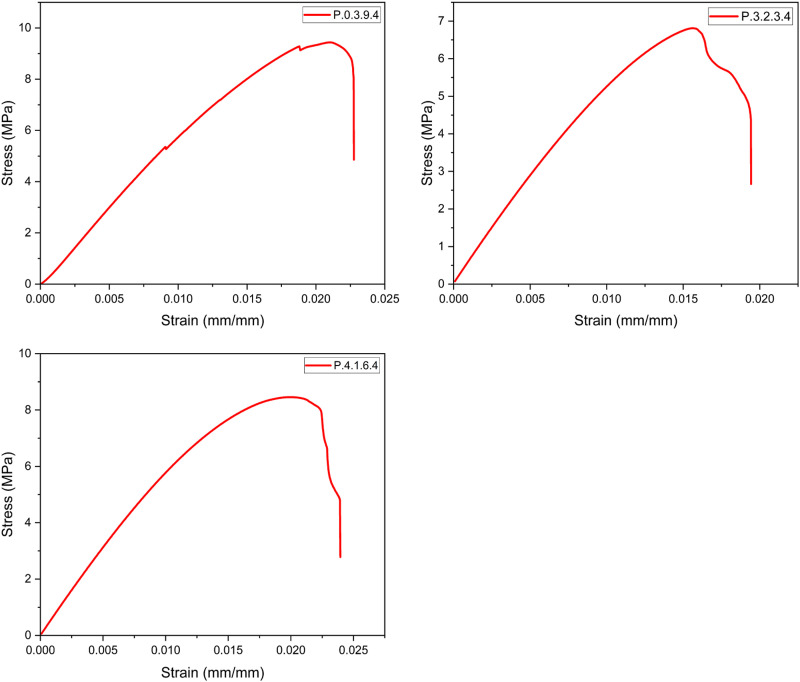
Stress-Strain analysis for specimens of 4th reprocessing cycle.

Once all the required specimens were printed, tensile testing was carried out. The stress-strain curves were plotted as shown in Figures 13, 14, 15, 16, and the Ultimate Tensile Strength (maximum stress in the stress-strain curve)^70^ was targeted. The Ultimate Tensile Strength (UTS) values of all the specimen combinations are shown in Tables 5 and 6. The tables also contain variance and the signal-to-noise ratio values, which will be used in the calculations for Taguchi Analysis.

**Table 5.** Tensile test results along with Taguchi Analysis 1.

Experiment number	A	B	C	D	Specimen	Ultimate tensile strength (MPa)			Taguchi analysis 1		
	RA	ID%	ET	RP		Sample 1 (T1)	Sample 2 (T2)	Sample 3 (T3)	Average (y_i_)	Variance (s_i_^2^)	Signal-to-noise ratio (SN_i_)
1	1	1	1	1	P.0.1.3.1	22.2	19.8	20.1	20.7	1.7	23.9
2	1	2	2	2	P.0.2.6.3	16.7	11.0	13.7	13.8	8.1	13.7
3	1	3	3	3	P.0.3.9.4	9.4	9.3	9.2	9.3	0.01	37.3
4	2	1	2	3	P.3.2.3.4	8.1	6.8	6.8	7.2	0.6	19.6
5	2	2	3	1	P.3.3.6.1	24.6	24.8	22.5	24.0	1.6	25.6
6	2	3	1	2	P.3.1.9.3	11.5	15.3	19.1	15.3	14.6	12.1
7	3	1	3	2	P.4.3.3.3	12.6	10.5	9.8	11.0	2.1	17.5
8	3	2	1	3	P.4.1.6.4	8.8	8.5	8.5	8.6	0.03	33.5
9	3	3	2	1	P.4.2.9.1	24.9	25.0	25.3	25.0	0.04	41.5

**Table 6.** Tensile test results along with Taguchi Analysis 2.

Experiment number	A	B	C	D	Specimen	Ultimate tensile strength (MPa)			Taguchi analysis 2		
	RA	ID%	ET	RP		Sample 1 (T1)	Sample 2 (T2)	Sample 3 (T3)	Average (y_i_)	Variance (s_i_^2^)	Signal-to-noise ratio (SN_i_)
1	1	1	1	1	P.0.1.3.1	22.2	19.8	20.1	20.7	1.7	23.9
2	1	1	1	2	P.0.1.3.2	12.2	18.0	11.3	13.8	13.2	11.6
3	1	2	2	1	P.0.2.9.1	25.9	27.2	26.5	26.5	0.4	32.6
4	1	2	2	2	P.0.2.9.2	23.5	19.6	23.7	22.3	5.5	19.6
5	2	1	2	1	P.4.2.3.1	18.4	17.9	24.9	20.4	15.3	14.3
6	2	1	2	2	P.4.2.3.2	18.5	10.9	10.4	13.3	20.7	9.3
7	2	2	1	1	P.4.1.9.1	24.4	24.4	23.5	24.1	0.3	33.6
8	2	2	1	2	P.4.1.9.2	22.6	23.9	18.3	21.6	8.8	17.2

From the results, it can be seen that the UTS of the specimens of first processing cycle was in the range of 20–26 MPa. This range of UTS reduced to 13–22 MPa for the second reprocessing cycle. While there was a significant drop to 12–16 MPa and 7–9 MPa for the third and the fourth reprocessing cycles, respectively. This mechanical degradation indicates the reducing recyclability of the material as the density as well as the tensile strength of PLA drops significantly in the course of four reprocessing cycles. The reduced strength of the material often leads to filament breakage during the spooling process of filament extrusion. This makes the recycling process les feasible. The color shifting property as already seen in Figure 11 gives a visual description of the deterioration of mechanical properties of PLA. This work open several scopes of work in the future related to the morphology and the chemical bond formations within a recycled material.

It was observed that specimens with higher infill densities had a better UTS when compared within a reprocessing cycle. This analysis was supported by,^71,72^ in which it has already been shown that higher infill densities result in better tensile strength. It was also observed that the UTS of the specimens was more for 0°/90° raster orientation,^35,73^ followed by 30°/−60° and 45°/−45° orientation. This analysis was supported by [192], [323], and [324], which showed that cross orientations (0°/90°) show better tensile strength than crisscross orientations (45°/−45°). Also, 30°/−60° has shown better tensile strength than 45°/−45°orientations for PLA samples. However, the results contradicted the work proposed in.^74^ It was deduced that for samples printed with 0.2 mm layer thickness, 30°/−60° orientation displayed maximum UTS followed by the crisscross and the cross orientations.

Lastly, it was observed that for the samples of a specific reprocessing cycle, Extrusion temperature (ET) had a direct relationship with the UTS. Samples at 220°C exhibited the highest UTS, followed by samples at 210° and 200°. This analysis was supported by,^31^ in which it was mentioned that for a temperature range of 200–220°C in PLA, ET has a direct relation with the tensile strength.

Here, it is important to clarify that the combination of parameters was a result of the Design of Experiments and hence each specimen is an experiment of the Taguchi analysis. Every graph represents a unique experiment and a specific set of parametric combinations. The graphs cannot be classified on the basis of any one parameter and hence were not merged and were drawn separately.

Table 7 shows the UTS values of all the combinations analysed in this work. The combinations from both TA1 and TA2 analyses are arranged in decreasing order of their UTS values which shows the effect of recycling, infill density, raster angle and extrusion temperature on the tensile strength values.

**Table 7.** Conclusion for UTS values of specimens from TA1 and TA2 analyses.

Specimen type	Ultimate tensile strength (UTS) in MPa
P.0.2.9.1	26.5
P.4.2.9.1	25
P.4.1.9.1	24.1
P.3.3.6.1	24
P.0.2.9.2	22.3
P.4.1.9.2	21.6
P.0.1.3.1	20.7
P.4.2.3.1	20.4
P.3.1.9.3	15.3
P.0.2.6.3	13.8
P.0.1.3.2	13.8
P.4.2.3.2	13.3
P.4.3.3.3	11
P.0.3.9.4	9.3
P.4.1.6.4	8.6
P.3.2.3.4	7.2

### Taguchi analysis

As mentioned earlier, two Taguchi analyses (TA1 and TA2) have been conducted in this work to compare the impact of FDM printing parameters (RA, ID%, and ET) and recycling effect (RP) on the tensile properties of PLA dog bone specimens. Since Taguchi targets the mean and variance of the process performance characteristic, these values are tabulated in Tables 8 and 9. Also, to calculate the effect each of the four parameters has on the output, the SN values have also been tabulated in these tables for every type of specimen. Tables 8 and 9 show the results for TA1 and TA2, respectively.

**Table 8.** Taguchi Analysis I results.

Level	A (RA)	B (ID%)	C (ET)	D (RP)
1	25.0	20.3	23.1	30.4
2	19.1	24.3	24.9	14.4
3	30.8	30.3	26.8	30.1
∆	11.8	10.0	3.7	15.9
Rank	2	3	4	1

**Table 9.** Taguchi Analysis II results.

Level	A (RA)	B (ID%)	C (ET)	D (RP)
1	21.9	14.8	21.6	26.1
2	18.6	25.8	19.0	14.4
∆	3.3	11.0	2.6	11.7
Rank	3	2	4	1

Now, for finding the rank of the parameters, it is important to find the range value (∆) of all the parameters. ∆ for any parameter is defined as the difference between the maximum SN and the minimum SN value for that parameter.

Δ=max(SNi)−min(SNi)

Larger ∆ of a parameter signifies a larger impact on the process outcome. In other words, if the same change in signal is implemented to all the parameters, the one with the highest range would bring a more impactful change to the output variable which is targeted. From Table 8, it can be seen that the highest ∆ is for parameter D (RP), followed by RA, ID%, and ET. Hence for TA1, reprocessing cycle is the most influencing parameter on the tensile strength of PLA specimens, followed by the rater angle orientations, infill density, and the extrusion temperature.

Similarly, from Table 9, it can be seen that yet again, the highest range value is for parameter D (number of reprocessing cycles), which confirms that at least up to four reprocessing cycles, recycling has the most impactful effect on the tensile properties of PLA specimens when compared with printing parameters such as RA, ID%, and ET. However, ID% is the second most influencing factor this time, followed by RA and ET. This infers that this order of impact for the four parameters can vary if the analysis is done for different levels or different processing conditions.

### Observations on experiments (Time challenges)

**Figure 17.**
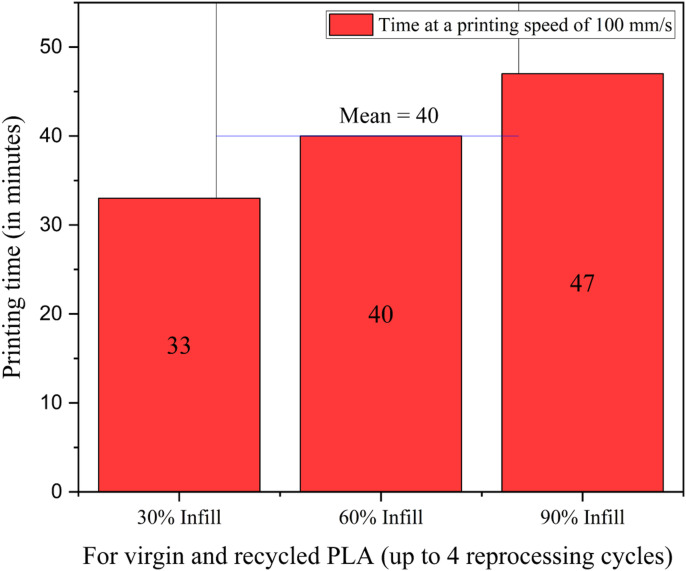
Results for time to print one specimen.

A systematic analysis has been done in this section to calculate the total number of specimens to be printed and the total number of days required to complete the printing based on the machine efficiencies, print time for a single specimen and machine as well as human tolerances. Based on the printing speed, which is 100 mm/s, the time taken for a single specimen to print is shown in Figure 17.

The 3D printing was conducted at the LIMDA lab at the University of Alberta from 10 am to 4 pm (an average of 6 hours duration) on all working days. Considering the time taken for a single specimen to print and the time slot available for printing in a single day, an average of 9 specimens were printed per working day. Table 10 shows the calculation for number analysis, whereas Table 11 shows the results of the time analysis for PLA specimens as per TA1.

**Table 10.** Calculation for number analysis for PLA specimens in TA1.

RA	ID, %	ET, °C	RP	Nomenclature	Specimens for tensile test	Specimens for next processing	Output required	Filament maker efficiency	Calculation for filament maker input	Filament maker input	Shredder efficiency	Calculation for shredder input	Input to be given
0°/90°	30	200	1	P.0.1.3.1	3	0	3	1	3	3	1	3	3
0°/90°	60	210	3	P.0.2.6.3	3	0	3	0.65	4.6	5	0.89	5.6	**6**
0°/90°	60	210	2	P.0.2.6.2	0	6	6	0.7	8.6	9	0.89	10.1	**11**
0°/90°	60	210	1	P.0.2.6.1	0	11	11	1	11	11	1	11	11
0°/90°	90	220	4	P.0.3.9.4	3	0	3	0.6	5	5	0.89	5.6	**6**
0°/90°	90	220	3	P.0.3.9.3	0	6	6	0.65	9.2	10	0.89	11.2	**12**
0°/90°	90	220	2	P.0.3.9.2	0	12	12	0.7	17.1	18	0.89	20.2	**21**
0°/90°	90	220	1	P.0.3.9.1	0	21	21	1	21	21	1	21	21
30°/−60°	30	210	4	P.3.2.3.4	3	0	3	0.6	5	5	0.89	5.6	**6**
30°/−60°	30	210	3	P.3.2.3.3	0	6	6	0.65	9.2	10	0.89	11.2	**12**
30°/−60°	30	210	2	P.3.2.3.2	0	12	12	0.7	17.1	18	0.89	20.2	**21**
30°/−60°	30	210	1	P.3.2.3.1	0	21	21	1	21	21	1	21	21
30°/−60°	60	220	1	P.3.3.6.1	3	0	3	1	3	3	1	3	3
30°/−60°	90	200	3	P.3.1.9.3	3	0	3	0.65	4.6	5	0.89	5.6	**6**
30°/−60°	90	200	2	P.3.1.9.2	0	6	6	0.7	8.6	9	0.89	10.1	**11**
30°/−60°	90	200	1	P.3.1.9.1	0	11	11	1	11	11	1	11	11
45°/−45°	30	220	3	P.4.3.3.3	3	0	3	0.65	4.6	5	0.89	5.6	**6**
45°/−45°	30	220	2	P.4.3.3.2	0	6	6	0.7	8.6	9	0.89	10.1	**11**
45°/−45°	30	220	1	P.4.3.3.1	0	11	11	1	11	11	1	11	11
45°/−45°	60	200	4	P.4.1.6.4	3	0	3	0.6	5	5	0.89	5.6	**6**
45°/−45°	60	200	3	P.4.1.6.3	0	6	6	0.65	9.2	10	0.89	11.2	**12**
45°/−45°	60	200	2	P.4.1.6.2	0	12	12	0.7	17.1	18	0.89	20.2	**21**
45°/−45°	60	200	1	P.4.1.6.1	0	21	21	1	21	21	1	21	21
45°/−45°	90	210	1	P.4.2.9.1	3	0	3	1	3	3	1	3	3

**Table 11.** Calculation for time analysis for PLA specimens in TA1.

Total specimens to be printed across 4 cycles	Rate of specimen printing (per day)	Calculation for days	Weekend days	Other holidays	Machine and human allowance	Sum of calculations	Number of days required
195	9	21.7	6.2	4	5	36.9	37

**Figure 18.**
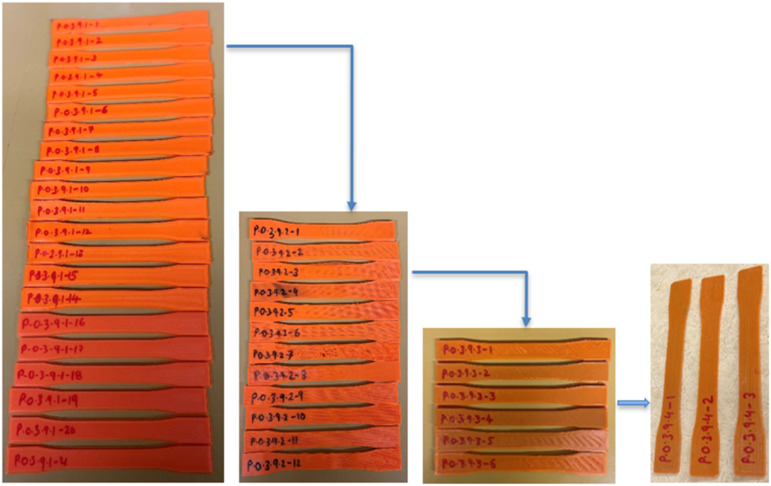
Number analysis for P.0.3.9.4 specimens.

The best way to understand the number analysis is to study the process in reverse order. For example, in Table 10, for conducting tensile tests on 3 specimens of P.0.3.9.4 combination, sufficient filament for 3 specimens should be obtained as an output from the filament maker. Hence, an efficiency of 60% for the filament maker at the fourth processing cycle means that shredded material of 5 specimens needs to be fed as input to the filament maker. On carrying forward this analysis, the shredder efficiency of 89% signifies that in order to obtain a shredded material output of 5 specimens, 5.618 or approximately 6 specimens need to be shredded. Hence, 6 specimens need to be printed as an output from the third processing cycle. Following the same concept and going into further reverse analysis, it can be observed that for providing sufficient material to print 6 specimens, 12 specimens need to be shredded at the start of the third processing cycle. Hence, 12 specimens need to be printed at the end of the second processing cycle. Again, to provide sufficient material to print 12 specimens, 21 specimens need to be shredded at the start of the second processing cycle. Hence, 21 specimens need to be printed at the end of the first processing cycle. Now since the first processing cycle involves virgin material and does not involve any shredding or filament making, it means that 21 specimens need to be printed at the start of the first cycle. To conclude, to test the required 3 specimens of P.0.3.9.4 combination, 21 specimens must be printed at. Similarly, for all the combinations, the numerical analysis is done based on the efficiencies of the equipment. The example of P.0.3.9.4 combination can be seen in Figure 18.

From Table 11, a total of 195 specimens need to be 3D printed over four reprocessing cycles. On moving further, at a rate of 9 specimens/day, it takes 21.667 days to print all the specimens. However, in a practical scenario, it is not possible to use the machine daily. Also, it is always a good idea to include machine and human allowances so that both man and machine fatigue are taken into consideration. In this analysis, weekends are considered as non-working days, and around 25% of the total days have been excluded as machine and human allowances. A margin of 4 extra days has been considered since the workplace for conducting this research is a university laboratory, which must follow the University holiday closure. To sum up, it will take around 37 days (6 hours daily) to finish the job of printing 195 desired PLA specimens.

Similarly, for TA2, by following the same methodology as in TA1, it can be concluded from Tables 12 and 13 that it will take around 9 days to finish the job of 3D printing the required 48 PLA tensile specimens over two reprocessing cycles. Hence the total number of days utilized for 3D printing of all the specimens (TA1 + TA2) in this entire research work is 37 + 9 = 46 days, which is approximately 1.5 months.

**Table 12.** Calculation for number analysis for PLA specimens in TA2.

RA	ID, %	ET, °C	RP	Nomenclature	Specimens for tensile test	Specimens for next processing	Output required	Filament maker efficiency	Calculation for filament maker input	Filament maker input	Shredder efficiency	Calculation for shredder input	Input to be given
0°/90°	30	200	2	P.0.1.3.2	3	0	3	0.7	4.3	5	0.89	5.6	6
0°/90°	30	200	1	P.0.1.3.1	3	6	9	1	9	9	1	9	**9**
0°/90°	90	220	2	P.0.2.9.2	3	0	3	0.7	4.3	5	0.89	5.6	6
0°/90°	90	220	1	P.0.2.9.1	3	6	9	1	9	9	1	9	**9**
45°/−45°	30	220	2	P.4.2.3.2	3	0	3	0.7	4.3	5	0.89	5.6	6
45°/−45°	30	220	1	P.4.2.3.1	3	6	9	1	9	9	1	9	**9**
45°/−45°	90	200	2	P.4.1.6.2	3	0	3	0.7	4.3	5	0.89	5.6	6
45°/−45°	90	200	1	P.4.1.6.1	3	6	9	1	9	9	1	9	**9**

**Table 13.** Calculation for time analysis for PLA specimens in TA2.

Total specimens to be printed across 4 cycles	Rate of specimen printing (per day)	Calculation for days	Weekend days	Other holidays	Machine and human allowance	Sum of calculations	Number of days required
48	9	5.3	1.5	1	1	8.9	9

However, it should be made clear that the time analysis is done in this work only considers the time for 3D printing of the specimens. The time for shredding and filament making is not included in this work as both the tasks were done simultaneously along with 3D printing of the specimens and hence did not add any extra time.

## Discussions

Several aspects of this work related to recycling effect on material properties, printing as well as machine parameters that have an impact on the DRAM process can be discussed in this section. In relevance to the product design and its mechanical properties, DRAM is yet to give concrete data as to how significantly a material degrades once it is recycled. This degradation varies according to the type of plastic and the number of reprocessing cycles. In this work, it was observed that PLA could degrade up to 75% in the course of four reprocessing cycles (P.0.2.9.1–2 = 27.2 MPa, whereas P.3.2.3.4–3 = 6.8 MPa). There was a change in color observed when the PLA material was recycled due to mechanical and thermal degradation. Another interesting analysis was the drop in weight of the specimens on subsequent recycling. This was due to the fact that the density of PLA was reduced on reprocessing, which resulted in a loss in weight as the volume of the samples was constant (ASTM D638 Type 1 standard). Hence, there is a scope of a huge database that can be created, including the type of plastic which actually can be recycled, its extent of recycling, and the change in mechanical properties it encounters during this recycling. In relevance to processing energy consumption, DRAM still fails to deliver information about the energy consumed in the 3D printing process under recycling. This is dependent on machine efficiencies as well as the recycled material properties. This work showed that the grinder and filament maker had efficiencies of around 89% and 60–75%, respectively, as per the reprocessing cycle. These efficiencies had an important role in determining the number of specimens to be 3D printed and the time taken to print them. Pre-known material properties such as temperature requirements at different reprocessing cycles can help determine the energy requirements for 3D printing of that recycled material. In relevance to the circular economy, DRAM lacks information about the life cycle assessment of the material. As mentioned earlier, there is a big literature gap in the context of how many times a particular plastic can be recycled. This work provides literature that PLA can be reprocessed at least four times, and there is a scope for recycling it even more. Adding to this, the effect of recycling on the printing parameters is yet to be explored completely. It is important to analyze the critical printing parameters for any recycled material. An attempt has been made in this work to rank the important parameters up to four reprocessing cycles for PLA.

## Conclusions

In this work, the Design of experiments via Taguchi analysis was conducted to reduce the amount specimens to be printed and analyzed. Due to this, it was possible to compare the recycling property (reprocessing cycles) with FDM printing parameters (raster angle, infill density, and extrusion temperature) up to three levels. Each of these properties had its own impact on the tensile strength of the specimens. For instance, as the number of reprocessing cycles increased, the tensile strength of the PLA samples decreased significantly. There was a drop of 33% in the tensile strength for 30% infill density samples when moving from the first reprocessing cycle to the second reprocessing cycle. This drop decreased to 23% and then 30% for the third and fourth reprocessing cycles. Likewise, there was a significant drop in tensile strength for 60% and 90% infill density specimens with each subsequent reprocessing cycle. Now, within a specific reprocessing cycle, it was observed that Infill density had a direct relationship with the tensile strength of the specimens. In the first reprocessing cycle, the tensile strength increased to 15% when moving from 30% infill to 60% infill and further increased by around 8.7% from 60% to 90%. This trend in infill densities was common in all reprocessing cycles. Similarly, in the case of raster angle orientation, it was observed that at all the reprocessing cycles, 0°/90° orientation showed the best tensile strength, followed by 30°/–60° and 45°/–45° orientations. Lastly, there was a direct relation observed between Extrusion temperature and the tensile strength of the samples at each reprocessing cycle. On conducting the two Taguchi analyses, it was seen that reprocessing effect was the most critical parameter among the four. Whereas at two-level analysis, infill density emerged as the second most influencing factor, while at three-level analysis, raster angle was the second most impactful parameter. The extrusion temperature was the least critical parameter in both analyses. Lastly, the number and time analysis was conducted, which gave an idea of the number of specimens to be printed at the initial stages to reach the desired number of specimens at a later reprocessing stage. This analysis was dependent on the efficiencies of the grinder and filament maker as well as the speed of 3D printing the specimens. For this, a separate speed test was conducted, which is also a part of this work.
